# Chrysin-Loaded Micelles Regulate Cell Cycle and Induce Intrinsic and Extrinsic Apoptosis in Ovarian Cancer Cells

**DOI:** 10.3390/nano15171362

**Published:** 2025-09-04

**Authors:** Serife Cakir, Ummugulsum Yildiz, Turgay Yildirim, Omer Aydin

**Affiliations:** 1 Department of Biomedical Engineering, Erciyes University, 38039 Kayseri, Turkey; genkoksrf@gmail.com (S.C.); gulsum.ugy97@gmail.com (U.Y.); 2 NanoThera Lab, Drug Application and Research Center (ERFARMA), Erciyes University, 38039 Kayseri, Turkey; 3 Department of Chemical and Biomolecular Engineering, Vanderbilt University, Nashville, TN 37235, USA; turgay.yildirim@vanderbilt.edu; 4 Nanotechnology Research and Application Center (ERNAM), Erciyes University, 38039 Kayseri, Turkey; 5 Clinical Engineering Research and Implementation Center (ERKAM), Erciyes University, 38039 Kayseri, Turkey

**Keywords:** raft polymerization, nanoprecipitation, chrysin, cell cycle, apoptosis, ovarian cancer

## Abstract

Effective intracellular delivery for ovarian cancer therapy remains a significant challenge. We present chrysin-loaded p(MMA-co-DMAEMA)-b-(OEGMA-co-DMA), PMOD-Chr, a nanoparticle platform precisely engineered via RAFT polymerization for advanced therapeutic delivery. This multi-functional platform features a hydrophobic p(MMA) core encapsulating chrysin (Chr), a pH-responsive p(DMAEMA) segment for endosomal escape, and a hydrophilic OEGMA (Oligo(ethylene glycol) methyl ether methacrylate) shell functionalized for enhanced cellular affinity and systemic stability. The combination of OEGMA and DMA (Dopamine methacrylamide) block facilitates passive targeting of ovarian cancer cells, enhancing internalization. Nanoparticles prepared via the nanoprecipitation method exhibited ~220 nm, demonstrating effective size modulation along with high homogeneity and spherical morphology. In A2780 and OVCAR3 ovarian cancer cells, PMOD-Chr demonstrated significantly enhanced cytotoxicity, substantially lowering the effective IC_50_ dose of Chr. Mechanistically, PMOD-Chr induced a potent G2/M cell cycle arrest, driven by the upregulation of the CDK1/Cyclin B1 complex. Furthermore, the formulation potently triggered programmed cell death by concurrently activating both the intrinsic apoptotic pathway, evidenced by the modulation of Bax, Bcl2, and caspase 9, and the extrinsic pathway involving caspase 8. These findings emphasize that precision engineering via RAFT polymerization enables the creation of sophisticated, multi-stage nanomedicines that effectively overcome key delivery barriers, offering a highly promising targeted strategy for ovarian cancer.

## 1. Introduction

Ovarian cancer is among the most fatal gynecologic malignancies worldwide, largely because the disease is typically asymptomatic until advanced stages and rapidly acquires resistance to standard therapies [1,2]. During the past two decades, clinical management has expanded beyond cytoreductive surgery and platinum-based chemotherapy to include targeted agents such as poly (ADP-ribose)-polymerase (PARP) inhibitors and anti-angiogenic drugs [3]. Nevertheless, five-year survival remains below 50%, and most patients experience recurrence driven by chemo- and PARP-inhibitor resistance [4].

With more than 300,000 new cases diagnosed globally each year, the limitations of current regimens underscore an urgent need for safer, more effective therapeutic strategies [1,2]. Standard chemotherapeutics and targeted inhibitors suffer from systemic toxicity, low selectivity, and dose-limiting adverse effects, while tumor heterogeneity continuously fuels resistance mechanisms [5,6]. Immune-checkpoint blockade has yielded only modest and inconsistent responses in ovarian cancer, often requiring complex combinatorial schedules to achieve measurable benefit [7,8]. These shortcomings collectively highlight the need to repurpose bioactive compounds with multi-target anticancer properties and to optimize their delivery.

In recent years, several natural compounds—such as curcumin, resveratrol, quercetin, and berberine—have been explored for their anticancer activities in cancers including ovarian cancer, owing to their ability to modulate key pathways [9,10,11]. These bioactive agents offer low systemic toxicity and multi-targeted mechanisms of action, further encouraging the search for phytochemical-based nanotherapeutics [12,13].

Among the natural compounds gaining attention, chrysin (Chr), a flavonoid found in passionflower, propolis, and honey, has emerged as a promising candidate due to its diverse pharmacological profile [14,15]. In preclinical models, chrysin has demonstrated significant anticancer activity, notably through the induction of apoptosis and inhibition of cell proliferation [16]. Mechanistically, it modulates key oncogenic signaling pathways such as NF-κB [17], MAPK [18], and PI3K/Akt [19,20] pathways intimately involved in cell growth, survival, and inflammation regulation [21]. Furthermore, chrysin’s pro-apoptotic effects have been linked to the generation of intracellular reactive oxygen species (ROS), leading to oxidative stress and downstream activation of cell death mechanisms [22]. Additional studies have shown its role in disturbing calcium homeostasis and triggering mitochondrial dysfunction, including the loss of mitochondrial membrane potential, an essential step in initiating the intrinsic apoptotic cascade.

Despite its broad-spectrum anticancer potential, the clinical application of Chr is significantly limited by poor aqueous solubility, low bioavailability, and rapid metabolic inactivation through glucuronidation and sulfation [23]. These pharmacokinetic drawbacks restrict its systemic efficacy and pose major obstacles to its clinical translation as a standalone therapeutic agent [24,25].

To overcome these challenges, a wide range of drug delivery strategies—including polymeric nanoparticles, micelles, liposomes, and metal-based systems—have been developed to improve chrysin’s solubility, stability, and tumor-targeting efficiency. These platforms, such as PEGylated carriers, chitosan-coated nanoparticles, and solid lipid systems, not only prolong systemic circulation and protect against premature degradation but also enable combinatorial therapeutic functions like photothermal therapy or radiosensitization [21,26,27]. Mechanistically, chrysin-loaded nanoparticles have been shown to amplify anticancer efficacy through enhanced ROS generation, mitochondrial membrane depolarization, and activation of apoptosis-related signaling pathways involving Bax, Bcl2, caspases, HIF-1α, and SIRT3 [28,29,30].

In this study, we developed and evaluated a well-defined, biocompatible copolymer-based nanocarrier system synthesized via Reversible Addition–Fragmentation Chain Transfer (RAFT) polymerization for the targeted delivery of chrysin in ovarian cancer models. While previous studies have primarily focused on improving chrysin’s solubility and passive anticancer effects, our approach integrates precision polymer engineering with detailed mechanistic investigation of tumor cell responses. The resulting formulation (PMOD-Chr) exhibited significantly enhanced cytotoxicity in A2780 and OVCAR3 ovarian cancer cells, reducing the effective IC_50_ of free chrysin. Mechanistically, PMOD-Chr induced a robust G2/M cell cycle arrest through upregulation of the CDK1/Cyclin B1 complex and concurrently triggered both intrinsic (Bax, Bcl2, caspase 9) and extrinsic (caspase 8) apoptotic pathways. While previous work has explored various chrysin-loaded formulations, our approach provides novel molecular insight into RAFT-synthesized polymeric delivery systems for ovarian cancer treatment.

## 2. Materials and Methods

### 2.1. Synthesis

#### 2.1.1. RAFT Polymerization

**Figure 1:**
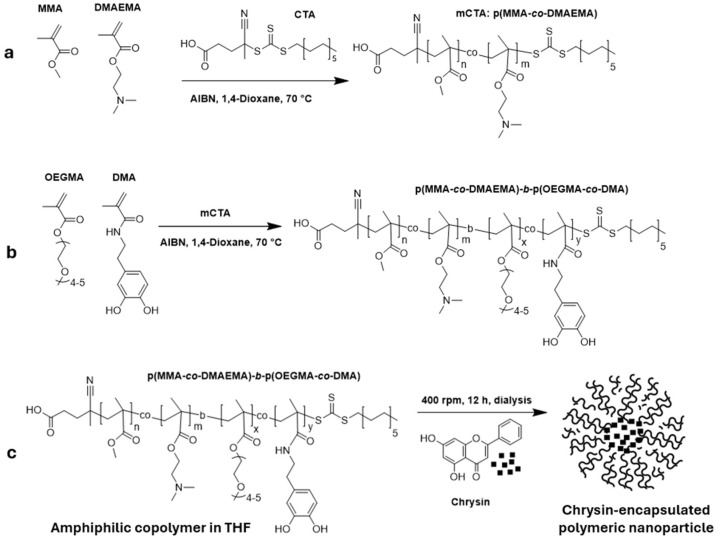
Schematic representation of PMOD-Chr nanoparticles synthesized via RAFT polymerization. (**a**) RAFT Polymerization of MMA and DMAEMA to synthesize p(MMA-*co*-DMAEMA) using and initiator, AIBN, and a chain transfer agent (CTA) in 1,4-dioxane at 70 °C. (**b**) Copolymerization of p(MMA-*co*-DMAEMA), DMA and OEGMA under RAFT conditions to form amphiphilic block copolymer p(MMA-*co*-DMA)-*b*-(OEGMA-*co*-DMA). (**c**) Self-assembly of the amphiphilic copolymer in aqueous medium in the presence of chrysin, forming chrysin-encapsulated polymeric nanoparticles via nanoprecipitation from THF.

p(MMA-*co*-DMAEMA) block copolymers (P1–P3) (PMDMs) were synthesized via RAFT polymerization in 1,4-dioxane using MMA (Methyl methacrylate), DMAEMA (2-(Dimethylamino) ethyl methacrylate), CTA (4-[[[(2-Carboxyethyl)thio]thioxomethyl]thio]-4-cyanopentanoic acid; Chain transfer agent), and AIBN (Azobisisobutyronitrile) (see Table 1 for ratios). The reaction proceeded under nitrogen at 70 °C for 11.5 h (Figure 1a). Reaction aliquots (t0 and tf) were collected for ^1^H NMR analysis. The polymers were purified by triple precipitation in n-hexane and redissolved in DCM (Dichloromethane).

p(MMA-*co*-DMAEMA)-*b*-(OEGMA-*co*-DMA) (PMOD) copolymers (BCPs) were synthesized via RAFT polymerization using OEGMA:DMA:CTA:AIBN at a molar ratio of 60:30:10:0.125 (Table 2). BCP1, BCP2, and BCP3 were synthesized using P1, P2, and P3 copolymers, respectively. Polymerization was carried out in 1,4-dioxane under nitrogen at 70 °C for 17 h with magnetic stirring (Figure 1b,c). The crude product was purified by triple precipitation in cold n-hexane, redissolved in DCM, and concentrated by rotary evaporation. Molecular weight and dispersity were determined by GPC (Gel Permeation Chromatography), and ^1^H NMR (Proton Nuclear Magnetic Resonance) confirmed compositional control.

#### 2.1.2. Synthesis of DMA Monomer

DMA was synthesized by reacting to methacrylic anhydride with dopamine HCl in a buffered aqueous solution under nitrogen [26]. After stirring at room temperature for 12 h, the mixture was extracted with ethyl acetate to remove non-polar impurities. Following acidification, the product was re-extracted, dried over MgSO_4_, filtered, and concentrated (Figure S1). Crude product was purified by repeated precipitation from ethyl acetate into cold n-hexane, then dried under vacuum. ^1^H NMR spectra were obtained in DMSO-d_6_ to confirm structure.

### 2.2. CMC Evaluation

The critical micelle concentration (CMC) of BCP3 was evaluated via Nile Red fluorescence assay [27]. A polymer stock solution (1.2 mg/mL in THF (Tetrahydrofuran)) was serially diluted (2.4 mg/mL to 0.000073 mg/mL), and micelles were formed by dropwise addition of 2 mL deionized water to each dilution, followed by THF evaporation over 48 h. Nile Red (10 µL of 0.1 mM in THF) was added to each sample and incubated for 2 h. Fluorescence intensity was measured at 550 ± 2 nm excitation and 620 ± 2 nm emission.

### 2.3. Nanoprecipitation of PMOD-Chr

Chrysin-loaded p(MMA-co-DMAEMA)-b-(OEGMA-co-DMA), PMOD-Chr nanoparticles were synthesized via a nanoprecipitation approach, based on solvent displacement and self-assembly principles [28,29]. Briefly, BCP3 (70 mg) and chrysin (7 mg) were dissolved in THF (7 mL), yielding 11 mg/mL stock. The nanoprecipitation method was applied: dropwise addition of the copolymer-Chr solution into 6 mL distilled water, followed by overnight stirring at 400 rpm (Figure 1c). Empty nanoparticles were prepared similarly. Solutions were dried by rotary evaporation (2 h), then diluted and analyzed by DLS (Dynamic Light Scattering). Final characterization included NTA (Nanoparticle Tracking Analysis) (after 100-fold dilution of 1 mg/mL samples) and SEM (Scanning Electron Microscope) (samples diluted to 0.01 mg/mL and further 100×).

A 1 mg/mL of Chr stock solution was prepared in DMSO. To determine the Encapsulation Efficacy (EE%) of PMOD-Chr nanoparticles, the nanoparticle solution was centrifuged at 15,000 rpm for 5 min to separate the PMOD from the supernatant containing unencapsulated Chr. The concentration of free Chr in the supernatant was then quantified by absorbance measurements at 351 nm using Synergy™ HTX Multi-Mode Microplate Reader (Agilent BioTek Instruments Inc., Winooski, VT, USA).

The amount of encapsulated Chr was calculated by subtracting the amount of free Chr in the supernatant from the initial amount of Chr used. The EE% was then determined using the following formula:

$$EE\%=\frac{weight\;of\;loaded\;Chr}{weight\;of\;initial\;Chr}\times 100$$

The Drug Loading Content (DLC) was calculated as the ratio of the weight of Chr encapsulated in the nanoparticles to the total weight of the nanoparticles, expressed as a percentage, using the following equation:

$$DLC\%=\frac{weight\;of\;encapsulated\;Chr}{total\;weight\;of\;polymer\;+\;Chr}\times \;100$$

### 2.4. Cell Culture

A2780 and OVCAR3, human ovarian cancer cell lines and HACAT human immortalized keratinocyte cell lines, were cultured in RPMI-1640 or DMEM supplemented with 10% FBS (Fetal Bovine Serum), 1% L-glutamine, 1% penicillin/streptomycin, and 1% amphotericin B, and incubated at 37 °C in a humidified atmosphere containing 5% CO_2_. The cells were seeded in flasks and monitored daily for viability and confluency.

### 2.5. Cell Proliferation Assay

IC_50_ (half maximal inhibitory concentration) values of Chr, PMOD, and PMOD-Chr in A2780 and OVCAR3 cancer cells and HACAT cells were determined. Five thousand cells were seeded in 96 wells and incubated for 24 h. A2780 and OVCAR3 cells were treated with treatment agents for 24, 48, and 72 h. Cell viability was determined using the MTT (3-(4,5-dimethylthiazol-2-yl)-2,5-diphenyltetrazolium bromide) assay. Cell viability was normalized by considering the untreated (control) well. IC_50_ values were calculated using GraphPad Prism 8 (Graphpad Software Inc., San Diego, CA, USA).

### 2.6. Colony Formation Assay

A2780 and OVCAR3 cells were seeded in 6-well plates at 1000 cells per well. The cells were incubated for 48–72 h to form colonies. Then, the cells were applied at 12 µM and 24 µM Chr, 12 µM and 24 µM PMOD, and 12 µM and 24 µM PMOD-Chr concentrations. After 7–10 days, when colonies of sufficient size were formed in the untreated wells, the cells were stained with 1% crystal violet in 10% methanol at room temperature for 30 min. The dye was removed, and the wells were washed three times with PBS. The number of colonies was counted. Then, the dye in the cells was dissolved with a 10% acetic acid solution and measured at 595 nm in a Synergy™ HTX Multi-Mode Microplate Reader (Agilent BioTek Instruments Inc., Winooski, VT, USA, ABD).

### 2.7. Apoptosis and Cell Cycle Assay

Cell cycle and apoptosis analyses of A2780 and OVCAR3 cells were quantified using BD FlowJoTM Software v10.10, Boston, MA, USA. Cells were seeded in 6 wells as 10 × 10^5^ cells/well and treated with Chr, PMOD, and PMOD-Chr at 12 μM and 24 μM concentrations for 48 h. Cells were collected after drug incubations. Cells were directly stained using the Biolegend 640914 Annexin V Apoptosis kit for apoptosis analysis. For the cell cycle assay, cells were fixed overnight using 70% ethanol at 4 °C. Fixed cells were incubated with RNase A solution at 37 °C for 30 min and then stained with PI (Propidium Iodide). Analysis was performed using a BD FAcs Canto II device. Graphs were obtained using FlowJo 7 (BD FlowJoTM Software v10.10, Boston, MA, USA) and GraphPad Prism 8 software (Graphpad Software Inc., San Diego, CA, USA, ABD).

### 2.8. Gene Expression Analysis

A2780 and OVCAR3 cells were grown in 25 cm^2^ flasks, and cells were treated with selected concentrations of Chr, PMOD, or PMOD-Chr for 48 h. After treatment, total RNA was isolated using Trizol to examine the effects on the mRNA expression levels of apoptosis-associated Bax, Bcl2, caspase 3, 8, and 9, and cell cycle-associated CDK1, CDK4, CDK6 (Cyclin-Dependent Kinases), Cyclin B1, and Cyclin D. Then, total RNA was obtained by Biorad cDNA kit (iScript™ cDNA Synthesis Kit, Biorad, CA, USA) for reverse transcription. The mRNA expression levels of target genes were analyzed by ABI StepOnePlus QPCR (Quantitative Polymerase Chain Reaction) device (Biorad Itaq, SYBR Green PCR Master Mix, Biorad, CA, USA) using cDNA Gene amplification. QPCR reaction conditions were initially set up at 95 °C for 10 min, followed by 45 cycles, each cycle consisting of 15 s denaturation at 95 °C and 1 min annealing/extension at 60 °C. Quantitative RT-PCR (Reverse Transcription Polymerase Chain Reaction) data were analyzed by the Comparative threshold (Ct) method, and fold inductions of samples were compared with untreated samples. Relative gene expression levels were assessed and normalized to β-Actin and GAPDH (Glyceraldehyde 3-phosphate dehydrogenase) housekeeping genes.

### 2.9. Statistical Analysis

All experiments were performed in triplicate, and data are presented as mean ± standard error of mean (SEM). Statistical significance was defined as a *p* value < 0.05. One-way ANOVA was used to determine the statistical significance of a variable in two or more samples, and two-way ANOVA was used to determine the statistical significance of two variables. Comparisons were made using Tukey’s post hoc multiple comparisons test and Dunnett’s post hoc multiple comparisons test. GraphPad Prism 8 (Graphpad Software Inc., San Diego, CA, USA) was used for graphing and statistical analysis.

## 3. Results

### 3.1. Characterizations of First Block Copolymers (Ps) as macroCTA Agent (mCTA)

**Figure 2:**
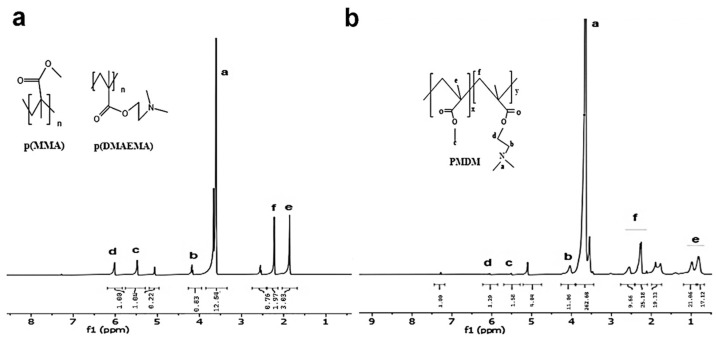
Representative ^1^H NMR spectra (recorded in DMSO-d_6_) of the reaction mixture at the initial stage (t0) and final state (tf) demonstrate the progress of the RAFT polymerization of the PMDM copolymer. (**a**) The MMA monomers exhibit distinctive vinyl proton signals at δ ≈ 5.5–6.2 ppm, attributed to the terminal -C=CH_2_ groups. Upon completion of the reaction, these signals are no longer detectable, indicating the full consumption of monomeric C=C bonds. (**b**) Broad new peaks emerge in the δ ≈ 0.8–2.2 ppm region, corresponding to the saturated polymethacrylate backbone (‘e’ and ‘f’). The continued presence of side-chain resonances, including protons ‘a’ [N(CH_3_)_2_] and ‘b’ [O–CH_2_–N–] from the DMAEMA unit, ‘c’ (OCH_3_ from MMA ester side chain), and ‘d’ (CH_2_ adjacent to ether or tertiary amine groups from OEGMA or DMAEMA units), confirms successful incorporation of the functional monomers into the polymer chain.

Details of the chemical reaction for the synthesis of copolymers P1, P2, and P3 by RAFT are illustrated in Figure S2. The transformation of the monomer within P1 and P2 is supported by key alterations in the ^1^H NMR spectra when comparing the *tf* to the initial state, t0. Specifically, the disappearance or significant attenuation of vinylic region signals (δ 4.5–7.0 ppm), characteristic of the monomer’s C=C double bond protons, along with the emergence of new, broader signals in the aliphatic region (δ 0.5–2.5 ppm), indicates the conversion of monomeric vinyl groups into a saturated polymer backbone. In the t0 spectrum (Figure S3a,c), peaks labelled t0-‘c’ (δ~5.6 ppm) and t0-‘d’ (δ~6.1 ppm) are attributed to vinyl protons, while t0-‘f’ (δ~2.0 ppm) indicates a methyl group. Peak t0-‘a’ (δ~3.5 ppm) is primarily assigned to the solvent. In the *tf* spectrum of P1 and P2 (Figure S3b,d), the notable persistence of peaks tf-‘c’ and tf-‘d’ (δ~5.5–5.9 ppm) suggests incomplete monomer conversion, despite the appearance of broad signals tf-‘e’ and tf-‘f’ (δ~0.8–2.5 ppm) corresponding to aliphatic protons and tf-‘b’ (δ~3.9 ppm) characteristic of side chains. In contrast, the ^1^H NMR analysis of the P3 copolymer provided compelling evidence of successful synthesis (Figure 2a). The characteristic monomer signals ‘c’ and ‘d’, present in the t0 spectrum (Figure 2b), were completely absent in the tf spectrum, signifying full monomer consumption and quantitative conversion into the P3 copolymer. Changes in peak morphology further supported copolymerization, consistent with the presence of macromolecules.

### 3.2. Characterizations of Second Block Copolymers (BCPs)

The block copolymers BCP1, BCP2, and BCP3 were synthesized via RAFT and exhibited high monomer conversions. GPC analysis revealed low Polydispersity Indices (PDIs) for all copolymers (Table 2), indicating good polymerization control and narrow molecular weight distributions. For BCP1, the theoretical (^1^H NMR) and measured (GPC) number-average molecular weights (*M_n_*) showed reasonable agreement (Table 2). However, BCP2 and BCP3 displayed significantly higher GPC-determined molecular weights compared to theoretical ^1^H NMR values, warranting further investigation. Nevertheless, BCP3, despite its highest M2 monomer content (30%), maintained high conversion and low PDI, suggesting successful synthesis of a well-defined copolymer with the targeted higher hydrophilic block content. The GPC-determined *M_n_* values consistently increased from BCP1 to BCP3 with increasing M2 monomer ratio.

Analysis of the GPC chromatograms (Figure S4) revealed distinct differences among the BCPs. Based on elution times, BCP2 eluted earliest, followed by BCP1, and finally BCP3. Peak widths indicated that BCP2 exhibited the narrowest peak, BCP1 showed an intermediate PDI, and BCP3 displayed the broadest peak. The largely monomodal main polymer peaks suggested successful block copolymer formation, with minor low molecular weight species potentially present in BCP1 and BCP3. BCP3 (70/30/1/0.125) was chosen for nanoparticle preparation due to its advantageous polymerization characteristics. It exhibited the highest monomer conversion rate (95.97%), which reflects efficient chain growth during RAFT polymerization. Furthermore, its molecular weight profile was optimal for forming uniform and stable nanoparticles: GPC analysis revealed a number-average molecular weight (*M_n_*) of 1.444 × 10^5^ g/mol and a weight-average molecular weight (*M_w_*) of 1.636 × 10^5^ g/mol, with a narrow dispersity index (*M_w_/M_n_* = 1.113) (Table 2). These features are critical for ensuring controlled drug loading and release behavior, making BCP3 the most suitable candidate for chrysin encapsulation and subsequent formulation development.

**Figure 3:**
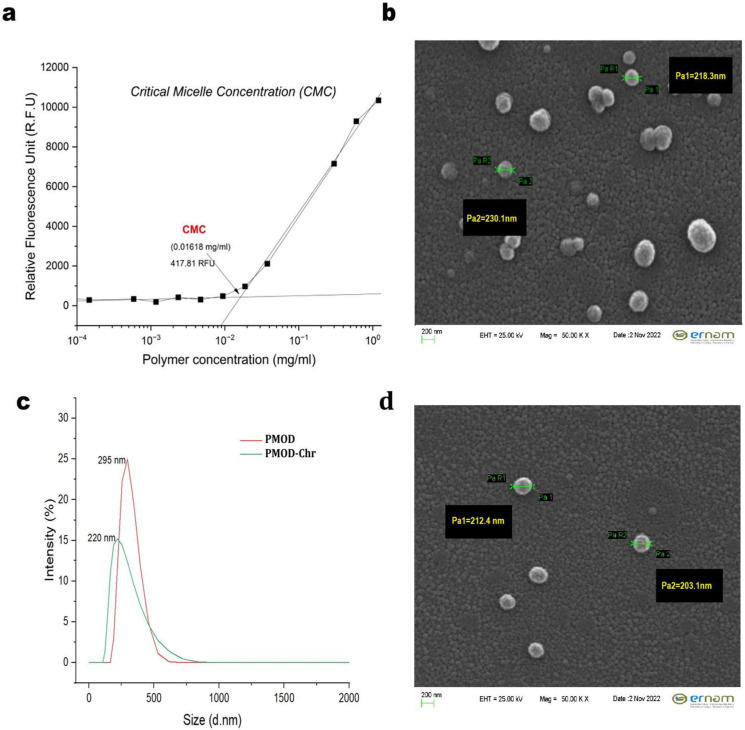
Chemical synthesis and characterization of self-assembling PMOD copolymer and PMOD-Chr nanoparticles. (**a**) Relative fluorescence units (RFUs) were measured across a range of polymer concentrations (mg mL^−1^). The CMC was determined to be 0.07416 mg/mL, corresponding to an RFU value of 214.89, as indicated in red on the graph. (**b**) PMOD-Chr surface morphology of the sample obtained via SEM. The image was acquired at an accelerating voltage (EHT) of 25.00 kV and a magnification (Mag) of 50,000×. The scale bar represents 200 nm. The measurements on the image indicate particle size (Pa). (**c**) Particle size distribution profiles from DLS measurements: PMOD nanoparticles (empty) and PMOD-Chr nanoparticles. (**d**) PMOD surface morphology of the sample obtained via SEM. The image was acquired at an accelerating voltage (EHT) of 25.00 kV and a magnification (Mag) of 50,000×. The scale bar represents 200 nm. The measurements on the image indicate particle size (Pa).

The CMC of BCP3 was determined to be 0.01618 mg/mL (Figure 3a). This relatively low CMC value signifies a high propensity for self-assembly and indicates considerable micelle stability against dilution, which is advantageous for biological applications.

The successful incorporation of DMA into the PMDM-OEGMA copolymer architecture was confirmed by ^1^H NMR spectroscopy (Figure S5). Characteristic resonance signals corresponding to the N-methyl protons of DMA were clearly observed at approximately δ 2.8–3.1 ppm. Integration of these signals, relative to specific proton resonances from the PMMA, DMAEMA, and OEGMA segments, quantified DMA incorporation, substantiating its covalent linkage within the copolymer structure.

### 3.3. Physicochemical Characterization of PMOD-Chr Nanoparticles

**Figure 4:**
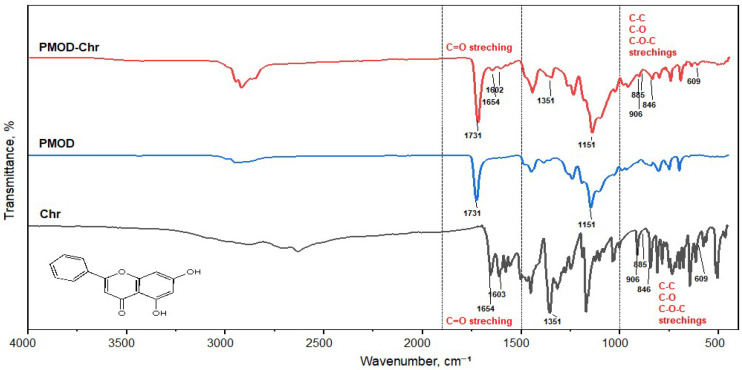
FTIR spectroscopy analysis to confirm the loading of Chr into PMOD. The spectra shown are for pure Chr (black), PMOD (blue), and PMOD-Chr (red). The Chr spectrum exhibits characteristic peaks for the C=O stretching vibration at 1654 cm^−1^ and aromatic ring vibrations at 1603 cm^−1^. The PMOD nanoparticle spectrum is dominated by a strong ester carbonyl (C=O) peak at 1731 cm^−1^ from the polymer backbone. The spectrum for the PMOD-Chr is a clear superposition of both components, retaining the polymer peak at 1731 cm^−1^ while also displaying the signature peaks of encapsulated Chr.

FTIR (Fourier Transform Infrared) spectroscopy confirmed Chr incorporation. Pure Chr (Figure S6) exhibited characteristic bands at ~1600 and 1580 cm^−1^ (aromatic C=C), ~1685 cm^−1^ (conjugated C=O), and above 3000 cm^−1^ (aromatic C-H). In the PMOD-Chr nanoparticles (Figure 4), while the matrix showed a strong C=O stretch around 1720–1725 cm^−1^, key Chr features persisted: aromatic C-H stretches above 3000 cm^−1^ and a shoulder at ~1600 cm^−1^. The shifted and less intense Chr carbonyl band, along with masked minor peaks, indicated Chr–matrix interactions and successful encapsulation.

DLS analysis revealed that the applied preparation protocol markedly influenced nanoparticle size (Figure 3c). Drug-loaded PMOD nanoparticles exhibited a reduced hydrodynamic diameter of approximately 220 nm, likely due to drug-induced polymer chain folding. This optimum small size is particularly advantageous for drug delivery applications due to EPR (Enhanced Permeability and Retention Effect), suggesting the protocol’s robustness in consistently producing compact nanoparticles.

NTA analysis corroborated the DLS findings, confirming the size distribution of the nanoformulation. PMOD-Chr demonstrated a more concentrated and stable distribution profile than empty nanoparticles. Post-processing analyses revealed an increased particle density within the same size range, highlighting the efficiency and reproducibility of the production process (Figure S7).

SEM showed generally spherical PMOD-Chr (Figure 3b) and PMOD (Figure 3d). Chr presence could affect morphology and aggregation, but the formulations often maintained spherical shape and homogeneity despite loading. These observations confirm the impact of formulation on nanoparticle characteristics.

### 3.4. Determination of EE (%) and DLC (%) of PMOD-Chr

Based on the drug amount used in the formulation (Figure S8) and subsequent calculations, the EE% of Chr into the nanoparticles was determined to be 57.96%. The total amount of drug loaded within the nanoparticles was found to be 5.79% of Chr, representing the DLC% for this preparation.

### 3.5. PMOD-Chr Inhibits Cell Proliferation and Clonogenic Survival in Human Ovarian Cancer Cells

**Figure 5:**
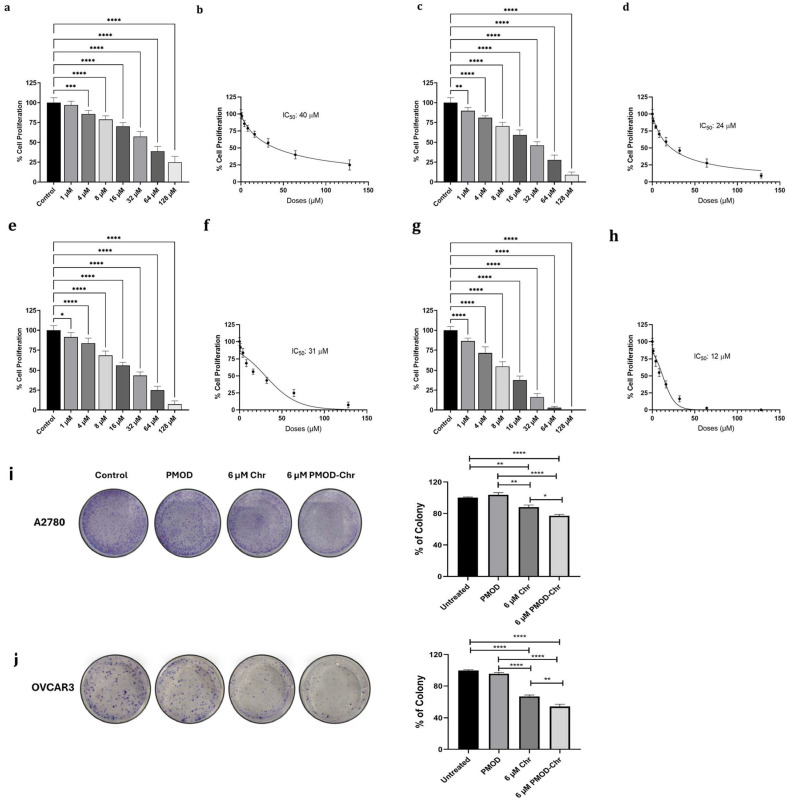
Showing the cell proliferation test results of Chr and PMOD-Chr in ovarian cancer cells. (**a**) Cell viability result of Chr in A2780 cells at 72 h and (**b**) IC_50_ dose at 72 h, (**c**) cell viability result of Chr in OVCAR3 cells at 72 h and (**d**) IC_50_ dose at 72 h, (**e**) cell viability result of PMOD-Chr in A2780 cells at 72 h and (**f**) IC_50_ dose at 72 h, (**g**) cell viability result of PMOD-Chr in OVCAR3 cells at 72 h and (**h**) IC_50_ dose at 72 h. Clonogenic survival of ovarian cancer cells was decreased by PMOD-Chr and Chr. (**i**) Colony formation of A2780 ovarian cancer cells and percent of colonies formed at each treatment. (**j**) Colony formation of OVCAR3 ovarian cancer cells and percent of colonies formed at each treatment. Each treatment was repeated 3 times, and results were displayed as the average ± SEM (standard error of the mean) of the three repeats and normalized to untreated groups. *p* value represents, *: *p* ≤ 0.05, **: *p* ≤ 0.01, ***: *p* ≤ 0.001, ****: *p* ≤ 0.0001.

The effects of Chr and PMOD-Chr on cell viability in the dose range of 1–128 µM in A2780 and OVCAR3 ovarian cancer cells were observed for 24, 48, and 72 h. The doses used in our study were calculated based on the Chr content of the nanomaterial. In two different ovarian cancer cells, doses of approximately 8 µM and above decreased viability at all time durations. In the comparison of Chr and PMOD-Chr, PMOD-Chr provided cell viability at lower doses, and this effect was more efficient in OVCAR3 cells (Figure 5a–h and Figure S9). It was seen that the designed PMOD without Chr did not affect cell viability (Figure S10). This showed that PMOD did not have a toxic effect in cells and that the designed nanoparticle could be an ideal cellular therapeutic carrier agent. On healthy human keratinocyte cells (HACATs), Chr showed toxic effects at doses of 32 µM and above, and PMOD-Chr only at a dose of 128 µM (Figure S11). PMOD-Chr has an effect on cancer cells; it only showed toxic effects on healthy cells at high doses. There was no significant change in colony numbers between the untreated and PMOD groups in A2780 and OVCAR3 cells. However, in both cell lines, the number of colonies decreased compared to the untreated cells with 6 µM of Chr and 6 µM of PMOD-Chr. In A2780 cells, a decrease of approximately 15% was observed when 6 µM Chr or PMOD-Chr groups were compared, and a decrease of approximately 25% was observed in OVCAR3 cells. Results showed that administration of PMOD-Chr resulted in increased inhibition of clonogenic survival of ovarian cancer cells (Figure 5i,j). 

### 3.6. PMOD-Chr Increased the Rate of Apoptosis Compared to Free Chr

Cells that do not undergo apoptosis are constantly dividing, and cancer occurs. Since the most basic feature of cancer is to escape apoptosis, anticancer drugs are required to lead the cells to apoptosis. Both ovarian cancer cells were stained with Annexin V-FITC (Fluorescein Isothiocyanate) and PI (Propidium Iodide) in order to examine the apoptotic feature with Chr and PMOD-Chr at 12 µM and 24 µM doses, and were examined by flow cytometry. In both cell lines, an increase in the rate of cells going to apoptosis was observed depending on the dose of Chr and PMOD-Chr.

**Figure 6:**
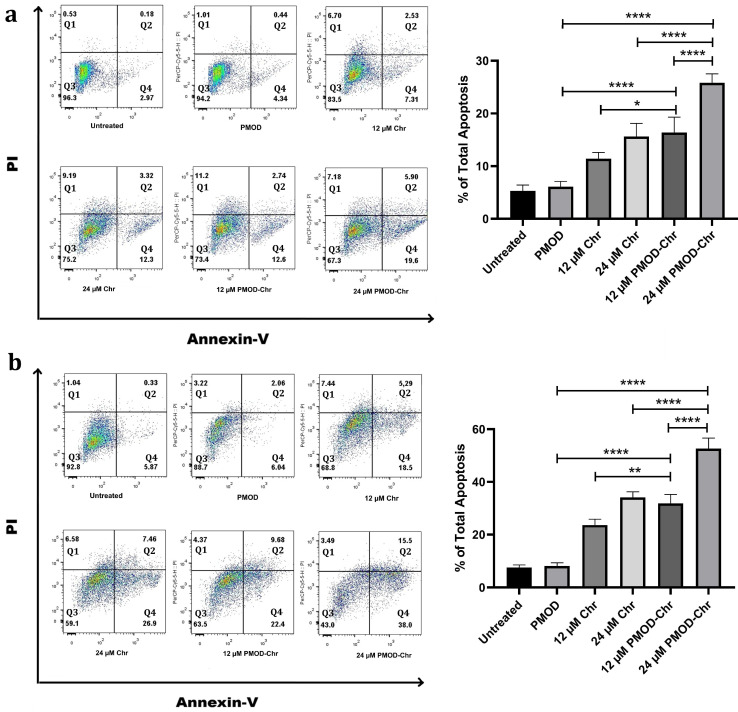

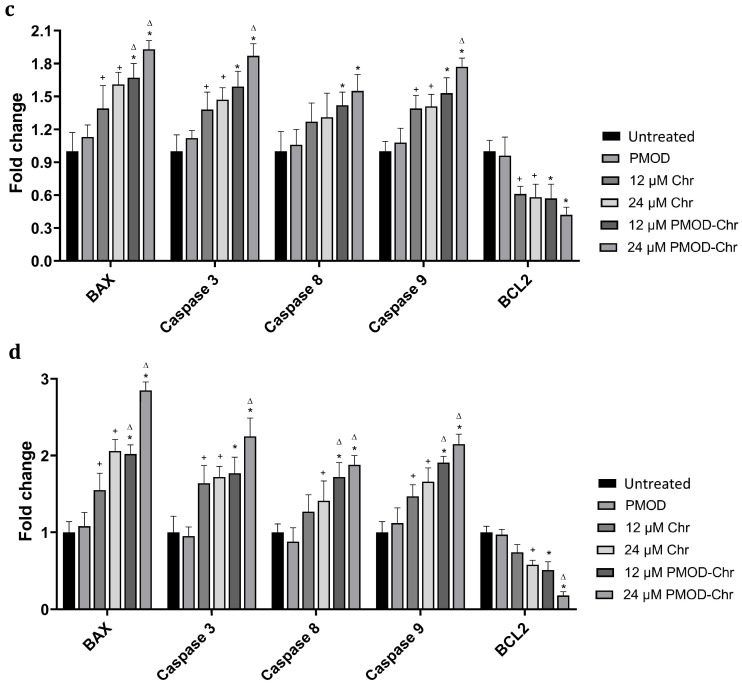
Analysis of apoptotic status of A2780 and OVCAR3 cells by flow cytometry and QPCR. (**a**,**b**): Flow cytometry analysis of A2780 (**a**) and OVCAR3 (**b**) cells treated with 12 µM and 24 µM free Chr and PMOD-Chr for 48 h, followed by staining with Annexin V-FITC and PI, and then flow cytometry analysis. PMOD-Chr and Chr significantly increased the apoptotic rate compared to untreated cells. The percentage of early and late apoptotic cells and necrotic cells is shown in the flow cytometry chart. The quandra in flow cytometry represent necrosis Q1 (Annexin^+^/PI^+^), late apoptosis Q2 (Annexin^+^/PI^+^), live Q3 (Annexin^−^/PI^−^), and early apoptosis Q4 (Annexin^+^/PI^−^) cells. Total apoptosis refers to all cells undergoing early (Q2) and late apoptosis (Q4). (**c**,**d**): RNA isolation and cDNA were obtained from A2780 (**c**) and OVCAR3 (**d**) cells treated with 12 µM and 24 µM Chr or PMOD-Chr for 48 h and apoptosis-related gene expression was examined. Normalization was performed with β-Actin and GAPDH housekeeping genes. Gene expression fold changes were obtained compared to control group cells.. Each treatment was repeated 3 times, and results were displayed as the average ± SEM of the three repeats and normalized to untreated groups. *p*-value represents in (**a**,**b**), *: *p* ≤ 0.05, **: *p* ≤ 0.01, ****: *p* ≤ 0.0001. *p* value represents in (**c**,**d**), +: *p* ≤ 0.05 compared to untreated group, *: *p* ≤ 0.05 compared to PMOD group, Δ: *p* ≤ 0.05 compared to Chr group.

As a result of the comparison of A2780 and OVCAR3 cells, Chr and PMOD-Chr were more effective in OVCAR3 cells at both 12 µM and 24 µM doses, and a high rate of apoptotic cell death was observed (Figure 6a,b and Figure S12). In both cell lines, the gene expression levels of Bax, Bcl2, caspase 3, 8, and 9 related to apoptosis of Chr and PMOD-Chr were examined by QPCR. In PMOD-Chr, A2780 and OVCAR3 ovarian cancer cells, proapoptotic or apoptotic Bax, caspase 3 and 9 gene expressions significantly increased and anti-apoptotic Bcl2 decreased (Figure 6c,d). In OVCAR3 ovarian cancer cells, it was observed that the intrinsic apoptotic pathway was activated with caspase 8 (Figure 6d). According to both ovarian cancer cells, PMOD-Chr most effectively leads to apoptosis in OVCAR3 cells, confirming the results obtained by flow cytometry.

### 3.7. Cell Cycle Is Arrested by PMOD-Chr in the Sub-G1 and G2/M Phase

**Figure 7:**
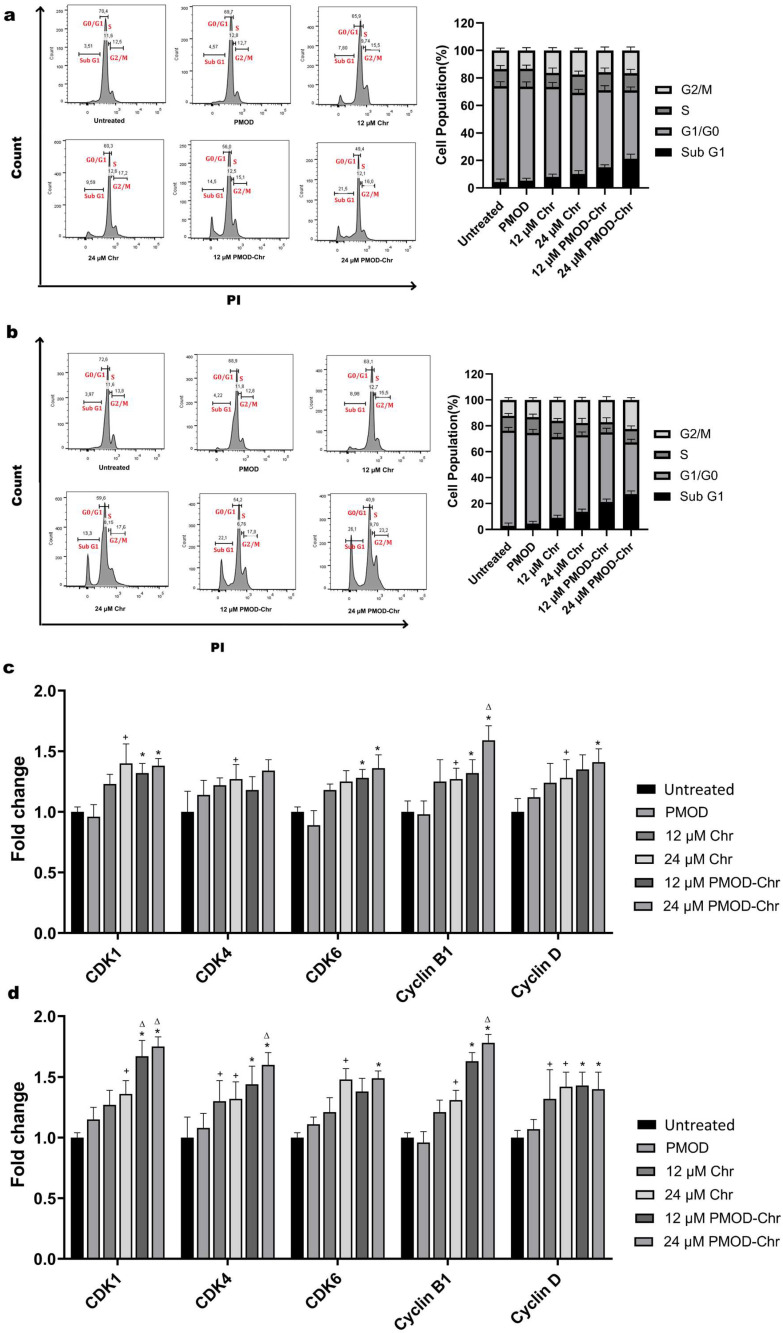
Effect of PMOD-Chr and Chr on DNA content in A2780 (**a**) and OVCAR3 (**b**) ovarian cancer cells. (**a**,**b**): Cells collected after 48 h of treatment with 12 and 24 µM Chr and PMOD-Chr were stained with PI. Cell cycle distribution was determined by flow cytometry analysis. Statistical charts of the cell population in G0/G1, S, and G2/M phases. (**c**,**d**): RNA isolation and cDNA were obtained from A2780 (**c**) and OVCAR3 (**d**) cells treated with 12 µM and 24 µM Chr or PMOD-Chr for 48 h, and cell cycle-related gene expression was examined. Normalization was performed with β-Actin and GAPDH housekeeping genes.. Each treatment was repeated 3 times, and results were displayed as the average ± SEM of the three repeats and normalized to untreated groups. *p* value represents; +: *p* ≤ 0.05 compared to untreated group, *: *p* ≤ 0.05 compared to PMOD group, ∆: *p* ≤ 0.05 compared to Chr group.

Cancer cells constantly divide and proliferate uncontrollably. Drug candidates to be used in cancer are expected to slow down or stop cell division. In our study, we examined the effect of PMOD-Chr on the cell cycle in two different ovarian cancers by staining the DNA content with PI using flow cytometry. In A2780 cells, PMOD-Chr allowed cells to remain in the Sub-G1 phase more than Chr. In OVCAR3 cells, PMOD-Chr was observed to increase cell accumulation in the G2/M phase as well as Sub-G1 (Figure 7a,b). This shows that PMOD-Chr not only leads to apoptosis with Sub-G1 in OVCAR3 cells but also stops cell proliferation at the mitotic stage by accumulating in the G2/M phase. Gene expression studies conducted with both cell lines provided regulation of CDK1, CDK4, CDK6, Cyclin B, and Cyclin D genes, which have important roles in the progression of the cell cycle. In A2780 cells, PMOD-Chr increased the expression of genes effective in G1 and G2/M phases. In OVCAR3 cells, when PMOD-Chr and Chr were compared, it upregulated the expression level of CDK1, and Cyclin B associated with the G2/M phase quite effectively (Figure 7c,d). When the results obtained by both flow cytometry and QPCR were examined, PMOD-Chr arrested the cell cycle in the Sub-G1 and G2/M phases more effectively in OVCAR3 cells.

## 4. Discussion

Ovarian cancer is the leading cause of gynecological cancer-related deaths [31,32]. One of the most important difficulties in the treatment of this disease is that drug use and options are limited due to side effects that occur as a result of drug use, toxic effects due to dose, systemic effects, and drug resistance [33,34]. In order to reduce this limitation and provide effective treatment options, targeted drug delivery strategies are used in treatments, and new systems to carry these drugs are needed [35]. The most current practical approach to overcome the limitations in the use of molecules that can be used in cancer treatment due to their hydrophobic properties and to protect the drug against corrosive reactions, destruction, and to increase its durability and effectiveness is to encapsulate these compounds in drug delivery systems [36].

Our rational nanoparticle design, precisely engineered via RAFT polymerization, centered on PMOD-Chr, leverages the pH-responsive behavior [37] of p(DMAEMA) [38] to overcome critical barriers in intracellular drug and gene delivery, particularly for challenges like ovarian cancer therapy. DMAEMA’s unique “switchable” nature, with a pKa around ~7.4 [39], dictates its hydrophilicity in acidic conditions and hydrophobicity in basic environments. This property allows precise control over nanoparticle morphology and size by simply adjusting ambient pH.

This intelligent design employs a dual mechanism to enhance therapeutic delivery [40]. Firstly, the OEGMA shell ensures high biocompatibility and prolongs systemic circulation by minimizing protein adsorption and immune recognition, promoting effective tumor accumulation [41]. Crucially, upon endocytosis into acidic endosomes, the PMOD block actively triggers the proton sponge effect [42]. This mechanism involves the dynamic protonation of the tertiary amine groups, leading to an osmotic influx of ions and water that swells and ultimately ruptures the endosomal membrane. This ensures the rapid and protected cytosolic release of encapsulated cargo, such as Chr in our ovarian cancer model, thereby preventing lysosomal degradation and maximizing therapeutic efficacy.

In the synthesis of novel polymeric micelles, RAFT polymerization proved instrumental in the successful creation of the P3 copolymer. Evidence of complete monomer transformation was compellingly derived from significant changes observed in the ^1^H NMR spectra. In the initial state’s (t0) spectrum, characteristic vinylic region signals at δ~5.6 ppm (t0-‘c’) and δ~6.1 ppm (t0-‘d’), attributed to the monomer’s C=C double bond protons, were clearly present. Crucially, in the final (tf) spectrum, these defining monomer signals were entirely absent, unequivocally indicating full monomer conversion and quantitative incorporation into the P3 copolymer. Concurrently, the emergence of new, broader signals in the aliphatic region (tf-‘e’ and *tf*-‘f’ at δ~0.8–2.5 ppm), corresponding to the saturated polymer backbone, along with a distinct signal at tf-‘b’ (δ~3.9 ppm), characteristic of side chains, further supported successful macromolecule formation. These findings collectively suggest that P3 was synthesized with a notably higher conversion efficiency, potentially leading to enhanced performance for its intended applications.

The CMC of our PMOD-Chr nanoparticles, determined by plotting polymer concentration against Nile Red fluorescence intensity, was found to be 0.01618 mg/mL (corresponding to a relative fluorescence unit of 417.81). This remarkably low CMC is a highly advantageous characteristic for drug delivery applications [43,44]. It signifies a strong propensity for the polymer to self-assemble into stable micellar structures and indicates that these nanoparticles can maintain their integrity and encapsulated cargo even under significant dilution in physiological environments, such as the bloodstream. This inherent stability directly enhances their potential as robust and effective drug delivery systems, preventing premature drug release and promoting targeted accumulation.

Characteristic resonance signals corresponding to the N-methyl protons of DMA were clearly observed at approximately δ 2.8–3.1 ppm. Integration of these signals, relative to specific proton resonances from the PMMA, DMAEMA, and OEGMA segments, quantified DMA incorporation, substantiating its covalent linkage within the copolymer structure [40]. In our PMOD-Chr nanoparticles, the DMA block functions as a crucial passive targeting agent for delivering Chr to ovarian cancer cells. Rather than active receptor binding, DMA exploits the unique characteristics of the tumor microenvironment; its inherently cationic nature enhances electrostatic interactions with the often negatively charged surfaces of ovarian cancer cells, promoting increased cellular affinity and internalization. This augmented cellular uptake, combined with the EPR effect [45] that passively accumulates nanoparticles in tumors, ensures more effective delivery of the therapeutic payload, Chr, to the target cells within the ovarian cancer tissue.

FTIR spectroscopy further confirmed the successful encapsulation of Chr within the PMOD nanoparticles. Pure Chr exhibited characteristic bands at ~1600 and 1580 cm^−1^ (aromatic C=C), ~1685 cm^−1^ (conjugated C=O), and above 3000 cm^−1^ (aromatic C-H). In the Chr-loaded nanoparticles, while the polymer matrix showed a strong C=O stretch around 1720–1725 cm^−1^, key Chr features persisted, including aromatic C-H stretches above 3000 cm^−1^ and a shoulder at ~1600 cm^−1^. The shifted and less intense Chr carbonyl band, alongside masked minor peaks, indicated interactions between Chr and the matrix, confirming successful encapsulation. This suggests that Chr is effectively retained within the nanoparticle matrix, potentially enhancing its bioactive benefits.

DLS analysis revealed that the nanoprecipitation method offers an optimized approach for nanocarrier design. Beyond in situ synthesis parameters, high-pressure homogenization further refines the physicochemical properties of these nanoparticles, critically influencing their therapeutic potential. While these specific cases sometimes exhibited slightly broader distributions, the overall size reduction is highly beneficial for improving in vivo pharmacokinetics by reducing premature clearance by the reticuloendothelial system and enhancing tumor accumulation through the EPR effect. While our nanoparticles slightly exceed the sub-200 nm threshold often cited for optimal tumor penetration, their hydrophilic OEGMA-rich corona and size profile still support efficient passive targeting. Ongoing efforts to modulate polymer architecture and formulation conditions aim to further enhance their therapeutic potential.

SEM consistently exhibits a spherical morphology; a common characteristic observed in nanoparticle systems and one that aligns perfectly with our production methodology. This spherical shape arises from the inherent tendency of the system to minimize its surface tension and achieve a thermodynamically more stable configuration. Synthesized via RAFT polymerization, our PMOD copolymers spontaneously self-assemble in an aqueous environment. During this self-assembly process, the hydrophobic PMMA core segregates from the aqueous phase to concentrate internally, while the hydrophilic OEGMA and pH-responsive DMAEMA blocks orient towards the particle’s outer surface. As this core–shell structure forms, the spherical geometry naturally represents the lowest free energy state at the molecular level. This observed spherical morphology offers significant advantages for both drug delivery and cellular uptake mechanisms. Spherical nanoparticles tend to exhibit a more uniform distribution within the body and can be more efficiently internalized by target cells via endocytosis.

Given the well-established proton sponge effect of p(DMAEMA), its inclusion in our nanoparticle design is expected to facilitate endosomal escape, thereby enhancing intracellular delivery efficiency. Although this mechanism was not directly quantified in our study, its contribution is supported by prior work demonstrating successful cytoplasmic release and bioactivity of delivered agents.

Chr-treated ES2 and OV90 ovarian cancer cells were observed to reduce cell proliferation. Chr has been studied in many different ovarian cancer cell lines, but there is no study on OVCAR3 cells [26,46,47,48]. A selenium-Chr polyurea dendrimer nanoformulation has been included in a study on the efficacy of OVCAR3 ovarian cancer cells, but only Chr was not examined [47]. Chr-loaded chitosan-folic acid-coated solid lipid nanoparticles were effective in pancreatic cancer, but Chr did not show much effect in ovarian cancer cells [26]. The IC_50_ value of Chr in A2780 cells at 72 h was seen to be 50 µM and above. In our study, it was found to be 40 µM. However, although the IC_50_ value was partially effective in PMOD-Chr, it was quite effective in OVCAR3 cells and reduced the IC_50_ value to 12 µM. According to these results, it is seen that PMOD-Chr inhibits cell proliferation more effectively than Chr alone. PMOD-Chr is non-toxic and can be a more effective targeted transport system.

Cell cycle checkpoints aim to ensure that the cell cycle progresses properly and healthily, and if the cell is not repaired, the cell will either stop itself or undergo a controlled death [49]. The Sub-G1 portion is the phase of the cell cycle where cells shrink in size and are more commonly identified as apoptotic cells [50]. In breast and prostate cancers, the Chr caused the cell cycle to remain in the Sub-G1 phase [51,52]. It has been reported in previous studies that Chr arrests A2780 cells in Sub-G1 of the cell cycle [48]. As a result of our cell cycle analyses performed with propidium iodide staining of A2780 and OVCAR3 cells, a greater accumulation in the Sub-G1 phase was observed in both PMOD-Chr groups compared to the other groups. This result shows that PMOD-Chr effectively drives the cells to the apoptotic pathway. Lim et al. revealed that Chr in urinary bladder cancer cells occurred in the G2/M phase of the cell cycle [50]. It gradually increased the percentage of cells in the Sub-G1 phase of the cell cycle in two different ovarian cancers. An increase in the percentage of G2/M phase cells was observed in ES2 ovarian cancer cells, but no change was observed in OV90 cells [22]. In our study with A2780 and OVCAR3 cells, an increase in the Sub-G1 phase was observed in the Chr and PMOD-Chr groups depending on the dose. The most dramatic increase was seen in the PMOD-Chr group in OVCAR3 cells at a dose of 24 µM. The G2/M checkpoint of the cell cycle prevents the cell from entering mitosis to repair the damaged genome and is involved in several pro-survival signaling pathways [53]. In the cell cycle study conducted with A2780 and OVCAR3 cells, an accumulation in the G2/M cell cycle was observed in the PMOD-Chr group. PMOD-Chr keeps cells in the Sub-G1 and G2/M phases in ovarian cancer, preventing the cells from completing mitosis or causing apoptosis, causing the cells to die via the apoptotic pathway.

The control mechanisms of cells, such as apoptosis, are one of the most important mechanisms in preventing normal cells from becoming cancerous. Apoptosis, which is the main mechanism of cancer cell death, is a programmed cell death process used by normal and cancer cells [54]. Chr initiates apoptosis, or cell death, in many cancer cells by regulating apoptotic cell pathways [55,56]. Lim et al. Chr has been shown to regulate the induction of apoptosis in ES2 and OV90 ovarian cancer cells via mitochondria-mediated intracellular Ca^2+^ accumulation. Chr triggered early and late apoptosis depending on the dose [55]. As reported in the literature, Chr has been reported to cause apoptosis in different ovarian cancer cells. The study has shown the effectiveness of Chr with two different cell types. In particular, it is observed that A2780 and OVCAR3 cell groups treated with PMOD-Chr, our nanocarrier produced by RAFT polymerization encapsulated in Chr, lead to cell death by apoptosis.

CDKs and cyclins serve as important control points in the cell cycle [49]. CDK4/6 and the cyclin complex are activated, especially in the G1 period of cells, and enable them to pass from G1 to S [57]. CDK1 and the cyclin B complex are activated towards the end of G2 and throughout the M phases of the cell cycle [58]. The complex of cyclin B-CDK1 migrates to the nucleus to activate several downstream pathways to allow cells to enter mitosis [56]. According to the results of cell cycle-related gene expressions, it is seen that PMOD-Chr increases gene activity related to the G2/M phase.

Among the master regulator genes involved in the activation or inhibition of apoptosis, the most important genes are proapoptotic BAX, antiapoptotic Bcl2, caspase 3/9 involved in intrinsic apoptosis, and caspase 3/8 involved in the extrinsic pathway [54]. In MDA MB-231 cells, Chr induced apoptosis by changing caspases 3 and 8 gene expression. Chr analog 8-bromo-7-methoxy Chr has been shown to induce apoptosis in A2780 cells [48]. According to the previous literature, Chr dose-dependently leads A2780 cells to apoptosis via Bax, Bcl, caspases 3, 8, and 9 [48]. In cells treated with Chr and Chr Niosome complex, decreased gene expression of apoptosis-related Bcl2 and increased gene expression of Bax and caspase 3 were observed [59]. As a result of gene expression studies conducted in A2780 and OVCAR3 ovarian cancer cells, it was understood that it regulates apoptosis-related genes and leads cells to apoptosis. PMOD-Chr, unlike Chr, activates intrinsic and extrinsic apoptosis and leads cells to apoptosis.

## 5. Conclusions

In this study, we developed a RAFT polymerization-based diblock copolymer nanocarrier (PMOD-Chr) for the targeted delivery of Chr in ovarian cancer cells. While free Chr exhibited moderate antiproliferative effects, PMOD-Chr significantly enhanced therapeutic efficacy at substantially lower doses. The formulation induced potent G2/M and Sub-G1 cell cycle arrest, particularly in OVCAR3 cells, and activated both intrinsic and extrinsic apoptotic pathways, highlighting its dual-action cytotoxic mechanism. These results demonstrate that precise molecular encapsulation via RAFT polymerization not only improves Chr’s bioavailability and intracellular delivery but also amplifies its antitumor activity. Our findings suggest that the PMOD-Chr platform offers a robust and versatile nanotherapeutic strategy with strong potential for further application across diverse cancer models and in vivo systems.

## Figures and Tables

**Table 1:** Molar ratios and masses of reagents used in the preparation of P1, P2, and P3 copolymers. P1, P2, P3—Block copolymers; MMA—Methyl methacrylate; DMAEMA—2-(Dimethylamino)ethyl methacrylate; CTA—Chain transfer agent; AIBN—Azobisisobutyronitrile.

	MMA	DMAEMA	CTA	AIBN
P1	3 g	471.63 mg	121.098 mg	6.84 mg
P2	3 g	1.179 g	151.372 mg	7.697 mg
P3	3 g	2.0122 g	0.1727 g	8.796 mg

**Table 2:** Selected characterization data of block copolymers OEGMA-DMA (M2): GPC analysis and comparison with theoretical ^1^H NMR data. M1—Monomer 1; M2—Monomer 2; CTA—Chain transfer agent; AIBN—Azobisisobutyronitrile; ^1^H NMR—Proton nuclear magnetic resonance; *M_n_*_—_Number-average molecular weight; *M_w_*—Weight-average molecular weight; PDI—Polydispersity index (*M_w_*/*M_n_*); Conv.—Conversion; Theo.—Theoretical.

	M1/M2/CTA/AIBN	Conv. [%]	*M_n_* (g mol^−1^), Theo. ^1^H NMR	*M_w_*	*M_n_*	*M_w_*/*M_n_* (PDI)
BCP1	90/10/1/0.125	89.4	11,017.67	1.324 × 10^4^	1.251 × 10^4^	1.058
BCP2	80/20/1/0.125	95.5	10,697.76	9.901 × 10^4^	8.205 × 10^4^	1.207
BCP3	70/30/1/0.125	95.9	10,645.75	1.636 × 10^5^	1.444 × 10^5^	1.113

## Data Availability

All data supporting the findings of this study are included in this published article and its Supplementary Materials.

## Supplementary Materials

The following supporting information can be downloaded at https://www.mdpi.com/article/10.3390/nano15171362/s1, Figure S1. Chemical reaction scheme illustrating the synthesis of dopamine methacrylamide (DMA) from methacrylic anhydride and dopamine hydrochloride., Figure S2. Chemical Reaction Scheme for the Synthesis of p(MMA-co-DMAEMA) (PMDM) First Block Copolymers (Ps). Schematic illustration of the RAFT polymerization process employed for the synthesis of the initial block copolymers P1, P2, and P3., Figure S3. 1H NMR Spectroscopy Analysis of First Block Copolymers P1 and P2. Representative 1H NMR spectra of copolymers P1 and P2 comparing the initial monomer mixture (t0, A and C, respectively) with the final polymer product (tf, B and D, respectively), indicating incomplete monomer conversion as evidenced by the persistence of vinylic proton signals. Figure S4. Gel Permeation Chromatography (GPC) Chromatograms of Block Copolymers (BCPs), BCP1, BCP2, and BCP3. RI (A) and LS (B) Overlays. Figure S5. 1H NMR spectrum confirming Dopamine Methacrylamide (DMA) incorporation into PMOD copolymer, Figure S6. Fourier Transform Infrared (FTIR) Spectrum of Pure Chr., Figure S7. Nanoparticle Tracking Analysis (NTA). Particle size distribution and intensity plots obtained from NTA for PMOD and PMOD-Chr nanoparticles, Figure S8. Calibration curve of Chr., Figure S9. Effects of Chr and PMOD-Chr on ovarian cancer cell viability, Figure S10. Effects of empty (PMOD) nanoparticle on ovarian cancer cell viability, Figure S11. Investigation of the toxic effects of Chr and PMOD-Chr on healthy human keratinocyte cells, Figure S12. Analysis of the effects of PMOD, Chr, and PMOD-Chr on early and late apoptosis.

## Abbreviations

The following abbreviations are used in this manuscript:

Chr	Chrysin
PMMA	Poly (Methyl Methacrylate)
DMAEMA	2-(Dimethyl Amino) Ethyl Methacrylate
OEGMA	Oligo (Ethylene Glycol) Methacrylate
PMDM	p(MMA-co-DMAEMA)
DMA	Dopamine Metacrylamide
PMOD	p(MMA-co-DMAEMA)-b-(OEGMA-co-DMA)
BCP	Block Copolymer
RAFT	Reversible Addition–Fragmentation Chain Transfer
EPR	Enhanced Permeability and Retention Effect
