# Supplementary material for: Chrysin-Loaded Micelles Regulate Cell Cycle and Induce Intrinsic and Extrinsic Apoptosis in Ovarian Cancer Cells
Source: Nanomaterials (Basel). 2025 Sep 4;15(17):1362. doi: 10.3390/nano15171362 (PMC12430720; doi:10.3390/nano15171362)
Supplement: Supplementary file 1 [file nanomaterials-15-01362-s001.zip › nanomaterials-3828482-supplementary.pdf]

Chrysin-Loaded Micelles Regulate Cell Cycle and Induce Intrinsic and Extrinsic Apoptosis in Ovarian Cancer Cells

Serife Cakir [1,2], Ummugulsum Yildiz [1,2], Turgay Yildirim [3] and Omer Aydin [1,2,4,5,*]

1 Department of Biomedical Engineering, Erciyes University, 38039 Kayseri, Turkey; genkoksrf@gmail.com (S.C.); gulsum.ugy97@gmail.com (U.Y.); omeraydin@erciyes.edu.tr (OA)
2 NanoThera Lab, Drug Application and Research Center (ERFARMA), Erciyes University, 38039 Kayseri, Turkey; genkoksrf@gmail.com (S.C.); gulsum.ugy97@gmail.com (U.Y.); omeraydin@erciyes.edu.tr (O.A.)
3 Department of Chemical and Biomolecular Engineering, Vanderbilt University, Nashville, TN 37235, USA; turgay.yildirim@vanderbilt.edu (T.Y.)
4 Nanotechnology Research and Application Center (ERNAM), Erciyes University, 38039 Kayseri, Turkey; omeraydin@erciyes.edu.tr (OA)
5 Clinical Engineering Research and Implementation Center (ERKAM), Erciyes University, 38039 Kayseri, Turkey; omeraydin@erciyes.edu.tr (OA)
* Correspondence: omeraydin@erciyes.edu.tr or biomer@umich.edu; Tel.: +90-352-207-6666 (ext. 32984)

Supplementary Data

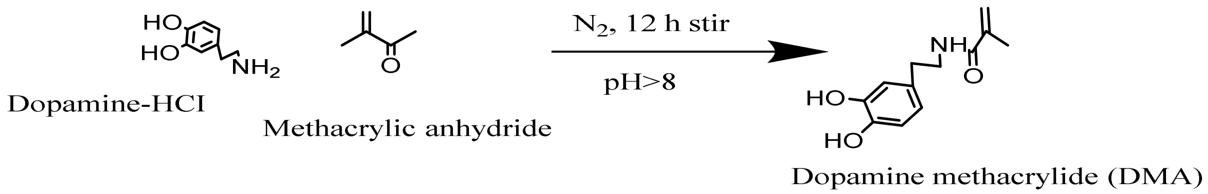

Fig. S1 Synthesis of Dopamine Methacrylamide (DMA) Monomer. This reaction scheme illustrates the stepwise synthesis of dopamine methacrylamide (DMA) via direct amidation of dopamine hydrochloride with methacrylic anhydride in a buffered aqueous solution under nitrogen atmosphere. Structural confirmation of the synthesized DMA monomer was performed using ^{1}H NMR spectroscopy in DMSO-d$_6$.

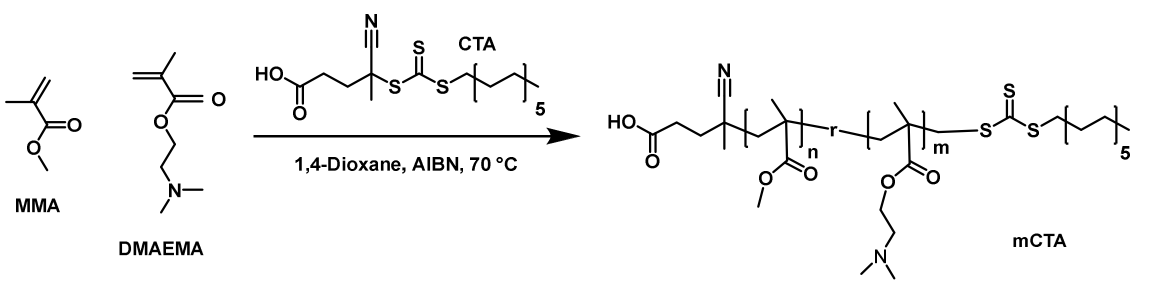

Fig. S2 RAFT Polymerization of p(MMA-co-DMAEMA) Block Copolymers (PMDMs). This reaction scheme illustrates the synthesis of p(MMA-co-DMAEMA) block copolymers, referred to as PMDMs, via RAFT polymerization in 1,4-dioxane. The polymerization was carried out using methyl methacrylate (MMA), 2-(dimethylamino)ethyl methacrylate (DMAEMA), a chain transfer agent (CTA), and azobisisobutyronitrile (AIBN) as the radical initiator. The reaction proceeded under a nitrogen atmosphere at 70 °C for 11.5 hours. Aliquots were collected at the initial (*t0*) and final (*tf*) time points for ^{1}H NMR analysis to monitor monomer conversion and polymer composition. The resulting polymers were purified by triple precipitation in cold n-

hexane and subsequently redissolved in dichloromethane (DCM) for further characterization. Structural confirmation of the synthesized mCTA was performed using ^{1}H NMR spectroscopy in DMSO-d_6.

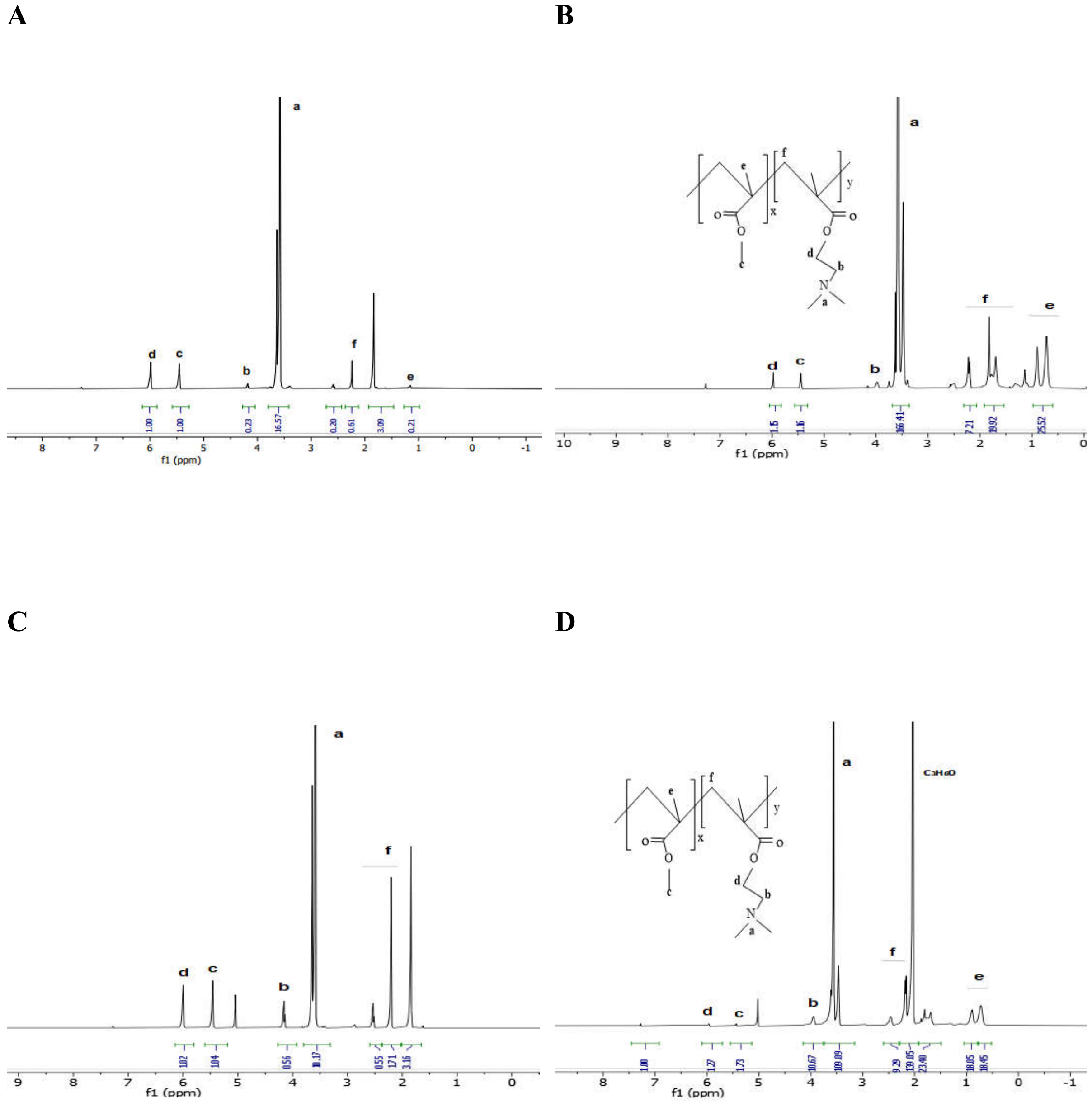

Fig. S3 Structural Confirmation of First Block Copolymers (P1 and P2, also referred as to PMDMs) via ^{1}H NMR Spectroscopy. Representative ^{1}H NMR spectra of p(MMA-co-DMAEMA) block copolymers (PMDMs) recorded in DMSO-d_6 confirm successful incorporation of both monomer units. Characteristic peaks corresponding to the methyl ester protons of MMA (~3.6 ppm) and the dimethylaminoethyl side chain of DMAEMA (~2.2–2.4 ppm) are clearly observed. The presence and relative integration of these signals validate the copolymer composition and support the efficiency of the RAFT polymerization process. Spectra were acquired for both initial and final reaction aliquots to monitor monomer conversion and assess polymer purity. ^{1}H NMR spectra comparing the initial monomer mixtures and final polymer products for copolymers P1 and P2. Spectra **A** and **B** correspond to P1 at the initial (*t0*) and final (*tf*) time points, respectively, while spectra **C** and **D** represent P2 at *t0* and *tf*. The persistence of vinylic proton signals ($\delta \approx 5.5$–6.2 ppm) in spectra **B** and **D** indicates incomplete monomer conversion despite extended polymerization. The emergence of broad polymer peaks and reduction of monomer-specific signals confirm partial formation of p(MMA-co-DMAEMA) block copolymers.

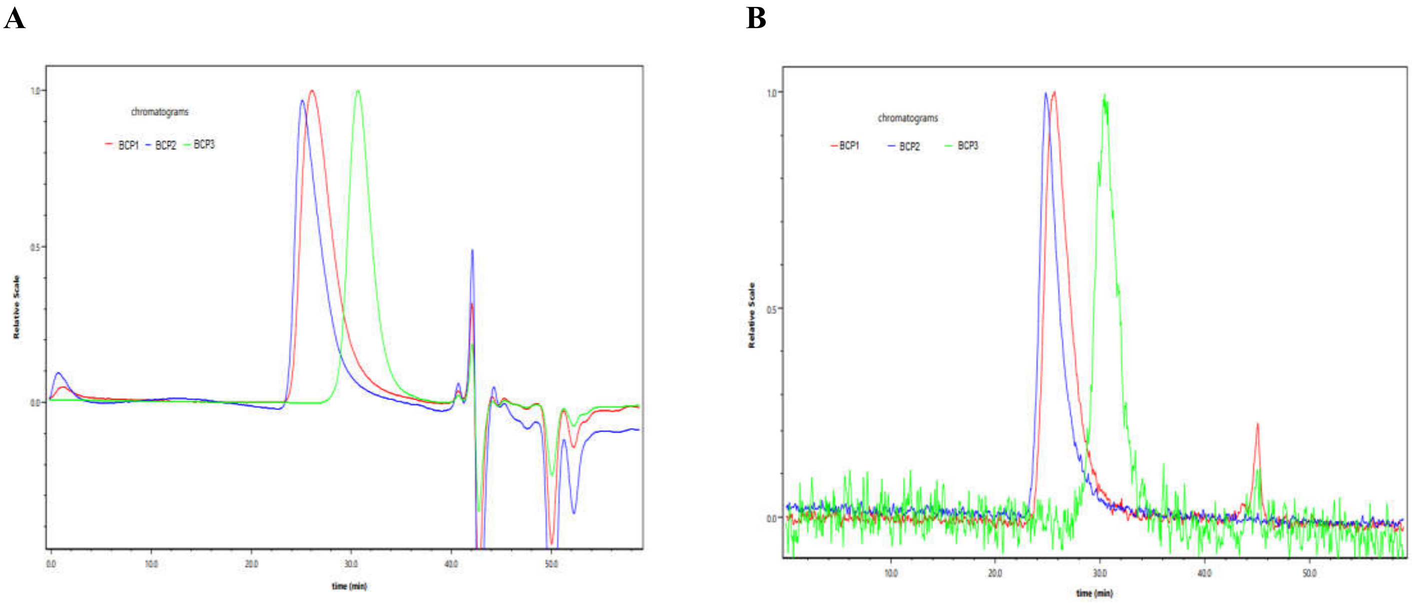

Fig. S4 Gel Permeation Chromatography (GPC) Analysis of p(MMA-co-DMAEMA)-b-(OEGMA-co-DMA) (PMOD) Block Copolymers. GPC chromatograms of synthesized p(MMA-co-DMAEMA)-b-(OEGMA-co-DMA) block copolymers (PMODs, hereafter referred to as BCPs) reveal successful polymer formation and molecular weight distribution. The elution profiles display unimodal peaks with distinct retention times, indicating controlled RAFT polymerization and consistent chain extension. Variations in peak position and width reflect differences in molecular weight and dispersity among the PMOD samples. These results confirm the efficiency and reproducibility of the block copolymer synthesis strategy. **(A)** RI (Refractive Index) and **(B)** LS (Light Scattering) Overlays.

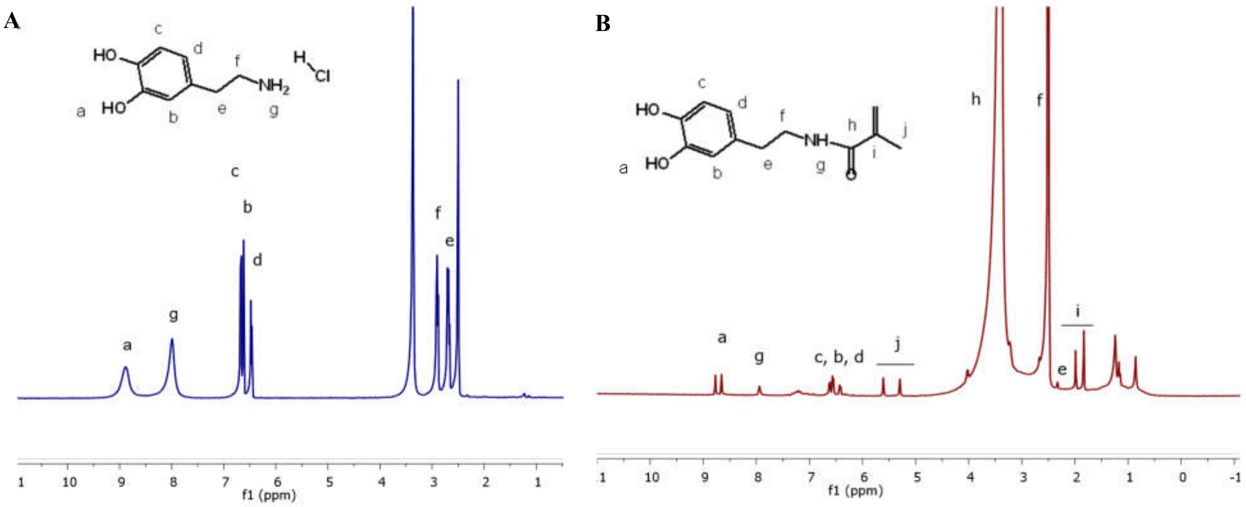

Fig. S5 ^{1}H NMR Spectroscopic Confirmation of Dopamine Methacrylamide (DMA) Incorporation into PMOD Copolymers. Representative ^{1}H NMR spectra comparing the initial monomer mixture (*t0*, spectrum **A**) and the final polymer product (*tf*, spectrum **B**) of the p(MMA-co-DMAEMA)-b-(OEGMA-co-DMA) (PMOD) copolymer recorded in DMSO-d_6 confirms the successful incorporation of the dopamine methacrylamide (DMA) unit into the polymer backbone. Characteristic aromatic proton signals of the catechol ring ($\delta \approx 6.5$–6.8 ppm) and methylene protons adjacent to the amide linkage ($\delta \approx 3.2$–3.4 ppm) are clearly observed, indicating the presence of DMA. ^{1}H NMR Signal Assignments: "a" aromatic H, catechol ring (DMA) ≈ 6.5–6.8 ppm; "b" CH_2–N, methylene adjacent to dimethylamino group (DMAEMA) ≈ 2.2–2.4 ppm; "c" CH_3–N, methyl protons on dimethylamino group (DMAEMA) ≈ 2.9–3.1 ppm; "d" CH_2–O, ethylene glycol methylene units (OEGMA) ≈ 3.6–3.8 ppm; "e" CH_3–COO, ester methyl group (MMA) ≈ 3.6 ppm; "f" CH_2 backbone, aliphatic methylene units (polymer backbone) ≈ 1.8–2.0 ppm; "g" CH_3 backbone, aliphatic methyl groups (MMA/DMAEMA) ≈ 0.8–1.2 ppm. The spectrum also displays signals corresponding to MMA, DMAEMA, and OEGMA segments, verifying the block copolymer composition. The integration of these peaks supports the structural integrity and targeted functionalization of the PMOD architecture.

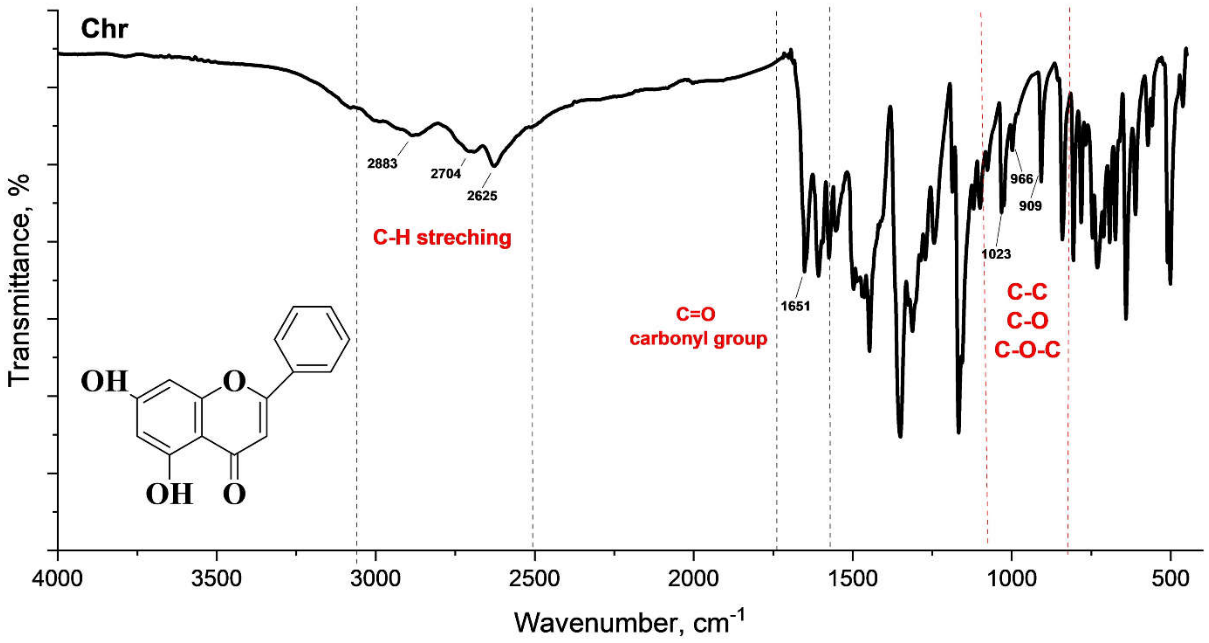

Fig. S6 Fourier Transform Infrared (FTIR) Spectral Profile of Chrysin (Chr). FTIR spectrum of Chrysin (Chr) confirms the presence of its characteristic functional groups. The sharp absorption band at ≈1650 cm⁻¹ corresponds to C=O stretching of the flavone backbone. Aromatic C=C stretching vibrations are observed at ≈1600 cm⁻¹ and ≈1500 cm⁻¹, consistent with the conjugated benzene rings. A broad band near ≈3400 cm⁻¹ indicates O–H stretching from phenolic hydroxyl groups. Peaks around ≈1250–1300 cm⁻¹ are assigned to C–O stretching vibrations, while bands in the ≈2850–2950 cm⁻¹ region reflect aliphatic C–H stretching. These spectral features collectively confirm the structural identity and purity of chrysin prior to its incorporation into polymeric systems.

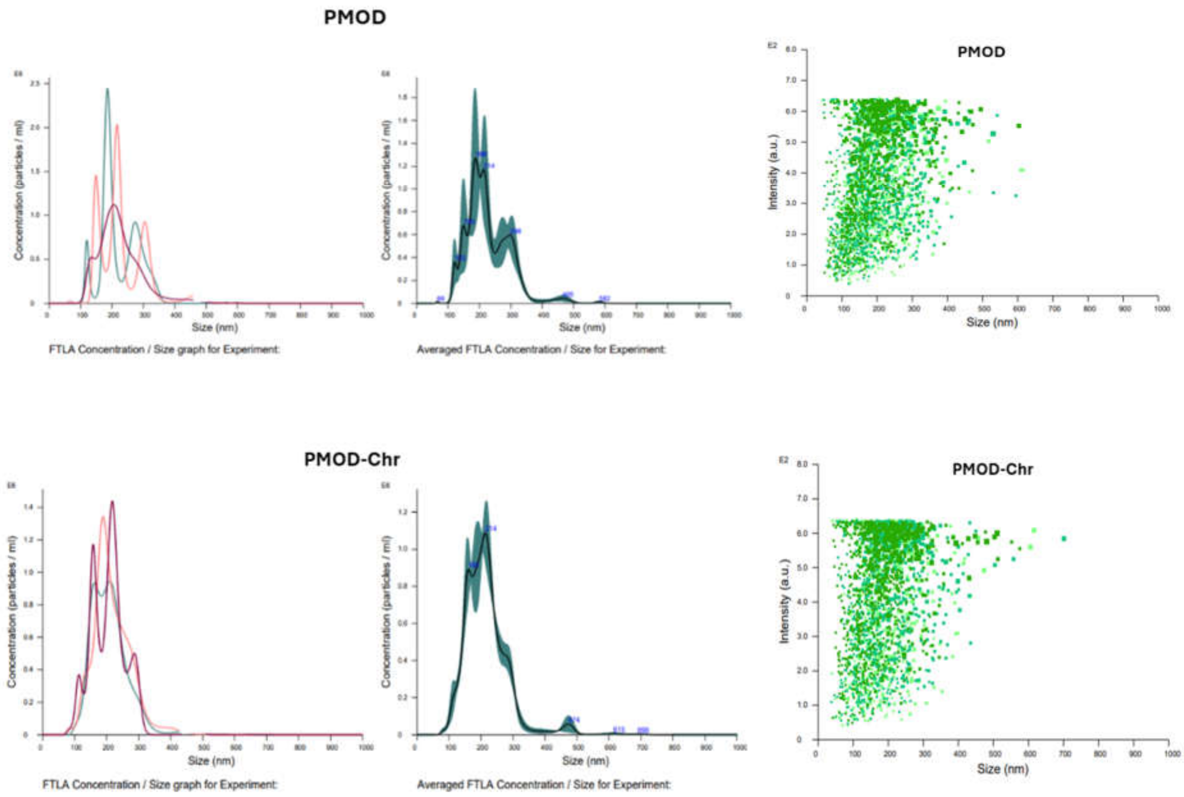

Fig. S7 Nanoparticle Tracking Analysis (NTA) of empty PMOD and PMOD-Chr nanoparticles. Particle size distribution and intensity plots obtained from NTA for PMOD and PMOD-Chr nanoparticles. Comparative size distribution and

concentration analysis of FTLA formulations over time. NTA profiles (left panels) depict nanoparticle size distribution at specified time intervals, highlighting formulation stability and aggregation tendencies. Corresponding bar graphs (right panels) represent mean particle sizes with standard error of the mean (±SEM), enabling quantitative assessment of formulation uniformity. FTLA: Functionalized Targeted Lipidic Assemblies.

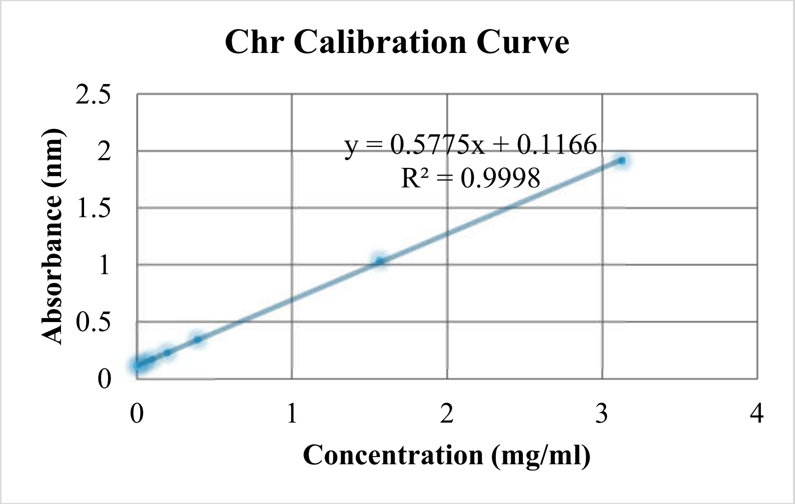

Fig. S8 Calibration curve of Chrysin (Chr). The curve was detected from serial dilution from 25 mg/ml to 0,02441 mg/ml by DMSO of Chr, y was expressed by absorbance average of the supernatant, x was expressed Chr concentration (mg/ml) at 351 nm absorbance from plate reader.

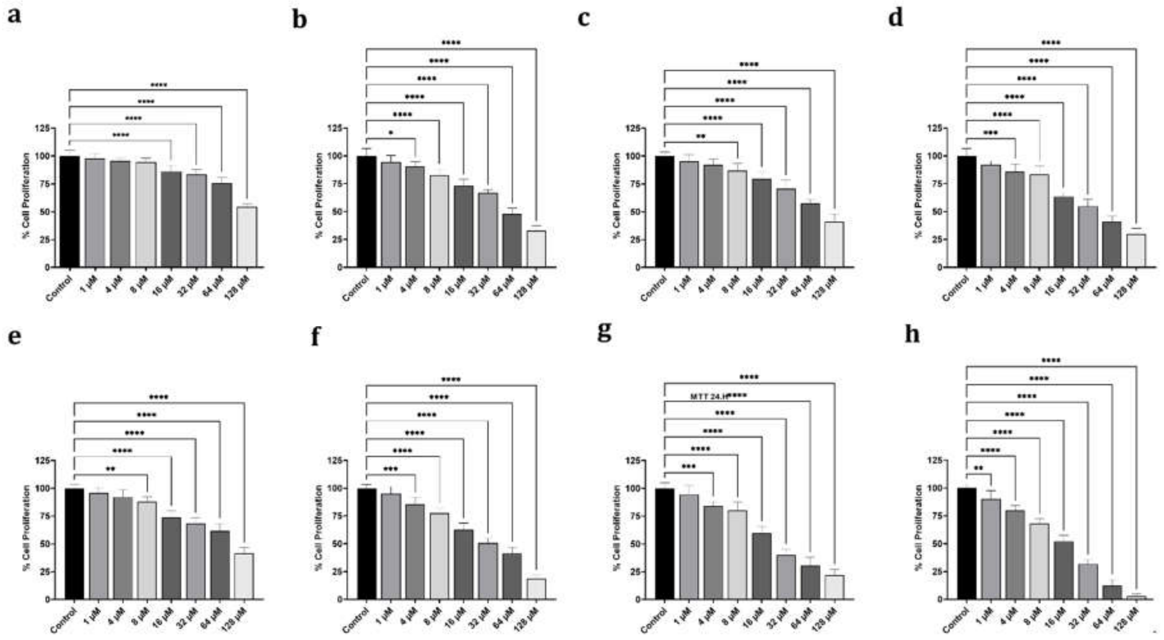

Fig. S9 Effects of Chr and PMOD-Chr on ovarian cancer cell viability. The effects of Chr and PMOD-Chr on cell viability of A2780 and OVCAR-3 ovarian cancer cell lines were examined at 24 and 48 hours. a) The effects of Chr on cell proliferation in A2780 cells at 24 hours, b) The effects of Chr on cell proliferation in A2780 cells at 48 hours, c) The effects of Chr on cell proliferation in OVCAR3 cells at 24 hours, d) The effects of Chr on cell proliferation in OVCAR3 cells at 48 hours, e) The effects of PMOD-Chr on cell proliferation in A2780 cells at 24 hours, f) The effects of PMOD-Chr on cell proliferation in A2780 cells at 48 hours, g) The effects of PMOD-Chr on cell proliferation in OVCAR3 cells at 24 hours, h) The effects of PMOD-Chr on cell proliferation in OVCAR3 cells at 48 hours, rs were evaluated by MTT assay. Each treatment was repeated

3 times and results displayed as the average ± SEM of the three repeats and normalized to control groups. p value represents ns: P > 0.05, *: p ≤ 0.05, **: p ≤ 0.01, ***: p ≤ 0.001, ****: p ≤ 0.0001.

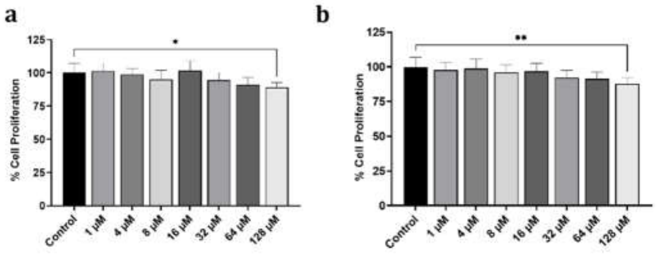

Fig.S10 Effects of empty(PMOD) nanoparticle on ovarian cancer cell viability. The effects of drug-free empty nanoparticle (PMOD) on cell viability of a) A2780 and b) OVCAR-3 ovarian cancer cell lines were investigated at 72 hours. The cytotoxic effects of the nanoparticles micelles at 72 hours were evaluated by MTT assay. Each treatment was repeated 3 times and results displayed as the average ± SEM of the three repeats and normalized to control groups. p value represents ns: P > 0.05, *: p ≤ 0.05, **: p ≤ 0.01.

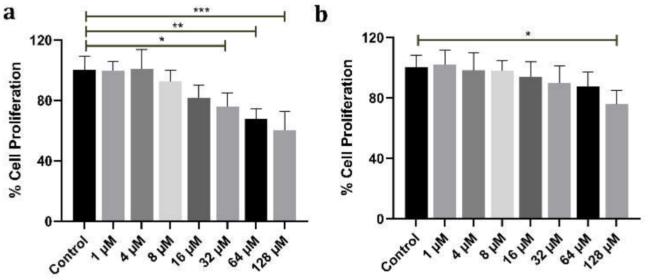

Fig.S11 Investigation of the toxic effects of Chr and PMOD-Chr on healthy human keratinocyte cells. The effects of a)Chr and b)PMOD-Chr on cell viability in HACAT human keratinocyte cells were examined at 72 hours. The cytotoxic effects of the formulations at 72 hours were evaluated by MTT assay. Each treatment was repeated 3 times and results displayed as the average ± SEM of the three repeats and normalized to control groups. p value represents ns: P > 0.05, *: p ≤ 0.05, **: p ≤ 0.01, ***: p ≤ 0.001.

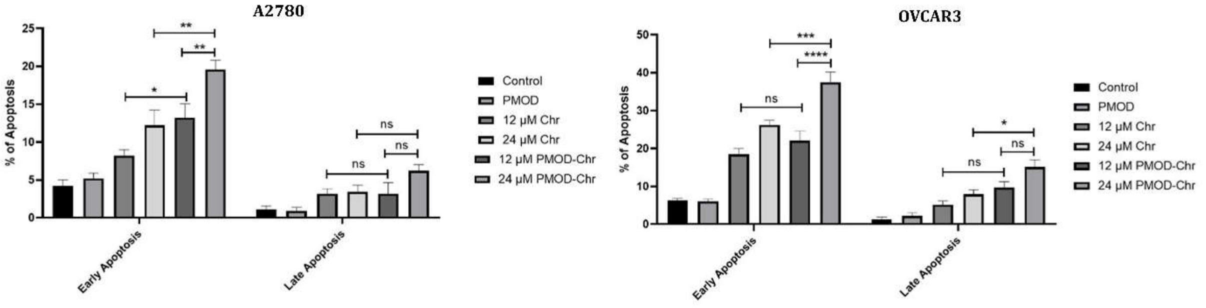

Fig. S12. **Analysis of the effects of PMOD, Chr and PMOD-Chr on early and late apoptosis.** A2780 and OVCAR-3 cells were treated with PMOD, Chr, and PMOD-Chr formulations at concentrations of 5, 10, 12, 25, 24, 50, and 100 µg/mL (corresponding to approximately 12 and 24 µM for Chr-based treatments) for 24 hours. Cell viability was assessed using the MTT assay. All formulations exhibited a dose-dependent reduction in viability, with PMOD-Chr showing the most pronounced cytotoxic effect at 100 µg/mL, reducing viability to 28.4% in A2780 and 31.7% in OVCAR-3. At intermediate doses (12 and 24 µM), viability dropped below 50%, indicating the onset of apoptosis. The steep decline at higher concentrations suggests progression from early to late apoptosis, consistent with apoptotic cell death mechanisms. These findings support the enhanced pro-apoptotic potential of the PMOD-Chr formulation. Each treatment was repeated 3 times and results displayed as the average ± SEM of the three repeats and normalized to control groups. p value represents ns: P > 0.05, *: p ≤ 0.05, **: p ≤ 0.01, ***: p ≤ 0.001, ****: p ≤ 0.0001.
